# Finding Our Bearings in Post-method Waters

**DOI:** 10.3389/fpsyg.2022.757684

**Published:** 2022-04-14

**Authors:** William Littlewood, Shufang Wang

**Affiliations:** ^1^Language Centre, Hong Kong Baptist University, Kowloon Tong, Hong Kong SAR, China; ^2^School of Languages and Communication Studies, Beijing Jiaotong University, Beijing, China

**Keywords:** post-method pedagogy, principles of communication, principles and dimensions of learning and teaching, engagement, memorization, communication

## Abstract

This paper proposes a framework to guide us in designing and implementing our classroom language pedagogy. It is based on three major principles which the teacher can keep constantly in mind: that the learners need to be engaged, that the language needs to be memorized, and that learning needs to move toward communicative competence. Each principle generates between two and four dimensions which the teacher can use to develop specific strategies.

## Searching for the “Right Method”

Language teaching nowadays is often described as being in a “post-method” stage. After the decades-long “search for the right method,” in which teachers have been urged to implement whatever method was officially supported (such as the audio-lingual method or situational language teaching) as the recipe for successful learning in every situation, it is now generally recognized that language learning is too complex, and there are too many differences in contexts, learners, and teachers, for us to find a “one-size-fits-all” solution. So we should base our work not on the prescriptions expressed in set methods but on more general “macro-principles,” which satisfy the fundamental requirements of language learning but can be implemented in ways that suit specific teaching situations. In other words, the principles themselves should be “context-free” but the ways of implementing them should be “context-sensitive” (Littlewood, 2014).

## Searching for Macro-principles

There have been several proposals for such macro-principles. Some proposals are derived primarily from the accumulated professional experience of teachers (e.g., Kumaravadivelu, 2006; Richards, 2006) whilst others are based mainly on what we know from research about how second languages are acquired (e.g., Ellis, 2005; Dörnyei, 2013). As one example, Dörnyei (2013) suggests that teachers should base their methodology on the principles that it should:

1. be meaning-focused and personally significant,
2. include controlled practice activities,
3. provide explicit initial input,
4. seek an optimal balance between implicit and explicit instruction,
5. recognize the importance of formulaic language,
6. provide exposure to large amounts of second language input, and
7. provide ample opportunities for genuine second language interaction.

For each macro-principle, the individual teacher can design specific classroom strategies and techniques suited to in his or her situation. For example, the first principle above is based on the general principle that language learning should be motivated by personal interest, but what is “personally significant” for (say) a group of adult learners will point the teacher to develop very different strategies from those for a group of elementary school learners.

## Clearing Away Myths From the Past

At the same time as searching for core principles, teachers have been keen to clear away some of the “myths” which have been handed down from the past, which have often been accepted without question but may obstruct teachers from designing their own approach. Here are some of the myths mentioned (and questioned) by Chia (2022) and Richards (2022): the use of the students’ mother tongue in the classroom is absolutely forbidden; exams and tests are an essential part of language learning; native speaker teachers are better than non-native speakers; we should be teaching British English or American English; anyone who can speak English can teach English; grammar is not a priority in communicative language teaching. Readers will be able to quote many other myths based on beliefs which were once widely accepted but are now either questioned or rejected completely.

## Developing a Framework to Guide Teaching

This article will work from three basic features of language learning and use them to develop a framework for classroom teaching which is both simple enough to guide our practice and rich enough to generate new ideas. These are the essential pillars on which the framework is based:

- Learning comes from engagement (since it is only through engagement that individuals connect with learning opportunities).
- Language must be memorized (otherwise new material will not be available for use beyond the immediate situation of learning).
- Language learning serves the requirements of communication (for most people, that is, the main source of their motivation).

The paper will now elaborate briefly on each of these pillars and then build them into a framework which, it is hoped, is based on principles which are not only clear and coherent enough to underpin teaching-in-action but also sufficiently generative to stimulate creativity and innovation. For the sake of clarity, the main components of the framework will be called “principles” and under each principle, we will distinguish between 2 and 4 “dimensions.”

### Principle 1: Learning Occurs Through Engagement

Engagement is obviously a key condition for learning to take place. How else would learning opportunities be processed and become meaningful? However, it is not uncommon to enter a classroom and find students who are paying little attention (or even none at all) to the learning opportunities that occur there.

Although the importance of engagement is obvious to the practicing teacher, it is only comparatively recently that the nature and conditions of engagement have been studied systematically. Based on recent work and their own analysis, Philp and Duchesne (2016) distinguish four important dimensions of engagement in the classroom:

- Behavioral engagement, e.g., the learners spend time on task and participate in the work.
- Social engagement, e.g., the learners are willing to listen to and cooperate with others.
- Emotional engagement, e.g., the learners feel motivation, enthusiasm, and enjoyment.
- Cognitive engagement, e.g., the learners pay sustained attention and try to make sense of what is new.

These dimensions are interdependent and intertwined. For example, social engagement leads naturally to the other three dimensions—this is one of the justifications for cooperative and task-based learning.

Each of these dimensions of engagement is also supported by the key factors which Keller (2010) includes in his influential “ARCS model of motivational design”:

- **A**ttention: arouse learners’ interest and learning curiosity, e.g., through novelty and variety: there are elements of the unusual or unexpected, as well as through authenticity: activities are associated with students’ own selves and interests.
- **R**elevance: satisfy the personal demands and targets of learners, e.g., through personalization: students link what they do in class with their lives outside it and through autonomy: students are allowed to make personal choices.
- **C**onfidence: assist learners in believing in their success and promote success, e.g., through emotional and intellectual safety: students feel free to take risks, as well as through relatedness: students feel socially connected to other classmates.
- **S**atisfaction: enhance achievement with rewards (internal and external), e.g., through learning that is supported by collaboration and sharing in a spirit of community challenge: students feel stimulated and rewarded by an acceptable degree of challenge.

### Principle 2: Language Must Be Memorized

If the new language material is not remembered, it will not be available for future use. Yet like engagement, memorization has also often been neglected in our discussions about pedagogy. In reviewing Bilbrough (2011), one of the few language teaching handbooks devoted specifically to memory activities, Maley (2013) suggests that this might be partly due to current preoccupations with communicative approaches and our association of memorization with rote learning techniques.

In an excellent “very short introduction” to memory, Foster (2009) reviews some dimensions which support memorization. These lie within the scope of the language teacher’s influence:

- Depth of processing

This is highlighted again and again as the key factor in memory. It refers to the level at which learners process new material when it is first encountered. Since the seminal work of Craik and Lockhart (1972), studies have consistently shown that the more meaningful the new material is and the more the whole person is engaged, the deeper and more lasting the memory traces are. For example, if new material is related to previous knowledge or to the learner’s own life and interests, it is remembered more strongly than, say, disconnected items which are encountered only in a superficial way. Studies also show that in general material is remembered better if it is related to everyday life, the concrete world, and the situations where it will need to be used.

Depth of processing is significant both for intentional memory (e.g., learning vocabulary for a test) and for incidental learning (e.g., subconscious acquisition in the course of communication).

- Practice

Studies have consistently demonstrated the importance of practice. Two important concepts for language teachers are “massed” practice and “distributed” practice. Studies have consistently shown that, given the same total learning time, practice which is distributed over a number of learning occasions separated by intervals produces stronger and more lasting memory (see the comprehensive synthesis in Cepeda et al., 2006). In a real classroom setting studied by Seabrook et al. (2005), “children whose teaching [in core literacy skills] consisted of three 2-min sessions per day showed more than six times the improvement of those who were taught for one 6-min session per day.” The implications for language teachers are obvious but in most classroom settings, of course, a series of short sessions requires more complex organization than one single massed session.

### Principle 3: Our Pedagogy Should Recognize a Continuum of Learning Activities From Form-Focused Work to Involvement in Communicative Interaction

The processes involved in the first two principles apply to all forms of learning: all learning requires memorization and engagement. For language teaching, we need to consider how to harness these processes in the service of learning to communicate. Thus, we need to assess how each activity contributes to learners’ communicative competence.

To facilitate this, we can conceptualize learning activities as below, in terms of a continuum from those which *focus on forms* to those which involve *communicative interaction*:

- Focus on forms

Activities which aim to develop the “part-skills” of communication may focus on the forms of language, e.g., formation of tenses or order of words, without any attention to meanings.

- Focus on forms and meanings

More often, an activity may focus not only on forms but also on the meanings that these forms convey, without yet engaging the learners in communicating messages. An example is the “question-and-answer” practice often used in situational language teaching.

- Focus on forms, meanings, and messages

The next stage in the development of communicative competence is when the learners also use these forms and meanings to communicate messages. Examples are the information gap activities and surveys which form an important component in communicative language teaching,

- Communicative interaction

The goal of language pedagogy is that learners use language creatively for expressing their own meanings, both in writing and in speaking, and for responding to the meanings of others. They engage in creative role-play, problem-solving, and free discussion. They have scope to express their own identities and the class becomes a community of learners. This is the goal of our teaching.

The rationale and nature of this continuum is explored in more detail in Littlewood (2011), where “communicative interaction” is further divided into “structured communication” and “authentic communication” to reflect different degrees of creativity.

## Summary of Principles and Dimensions in Classroom Language Teaching

**Figure 1:**
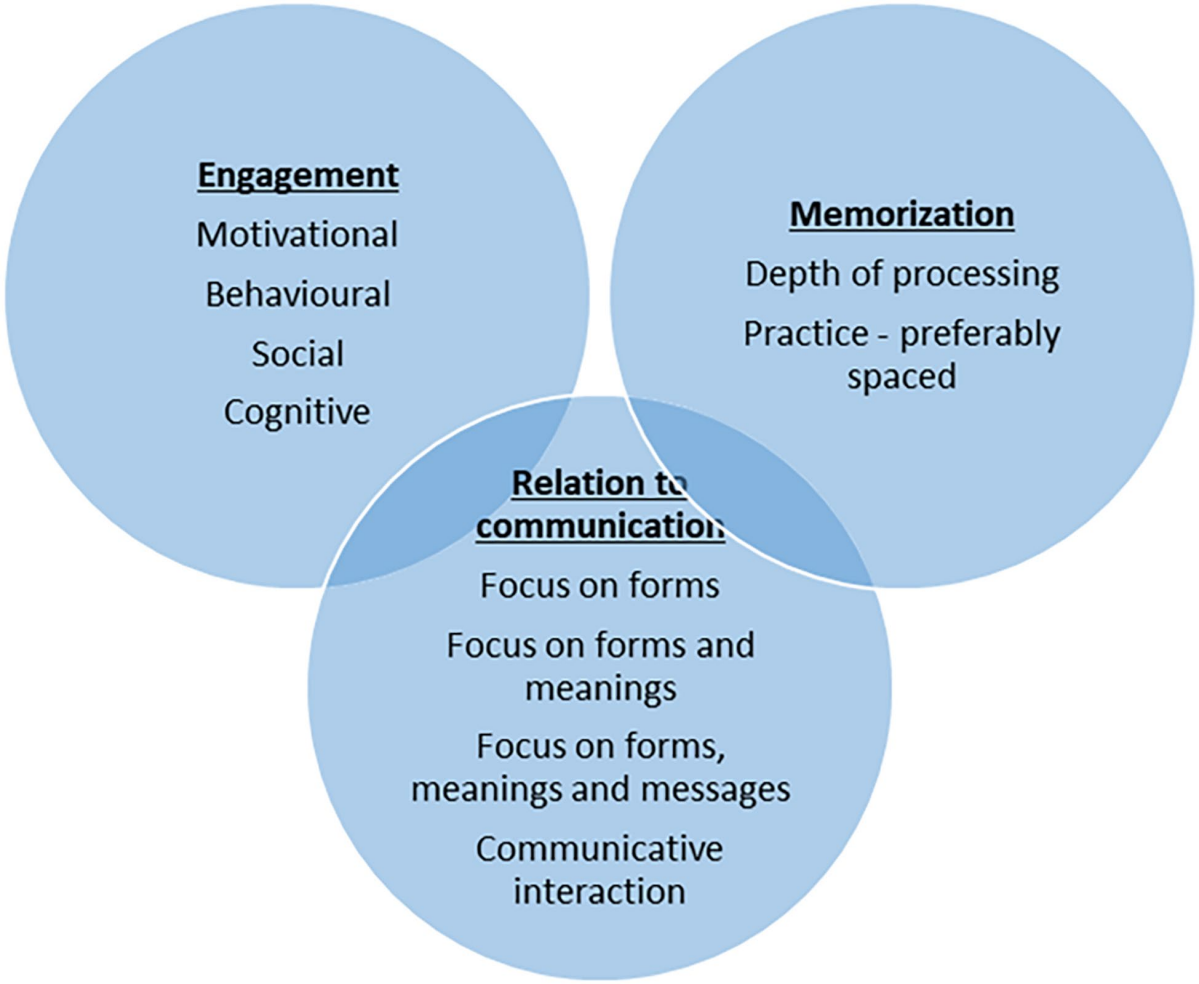
Principles and dimensions in classroom language teaching.

The 10 factors proposed above can be converted directly into macro-principles comparable to those of Dörnyei (2013) and the other authors mentioned above (e.g., “teachers need to attend to motivational engagement”; “learning occurs most effectively when there is a deep level of processing”; “teachers need to stimulate communicative interaction within a community of learners”). Alternatively, they can be situated as dimensions in a more fluid framework which allows us to better conceive them as operating simultaneously and interactively to form a coherent pedagogy. This is represented in Figure 1.

The three major principles govern every moment of our teaching: we constantly need to consider the extent and nature of our students’ engagement, the measures we and they can take to strengthen memorization, and the relationship of the classroom activities to the goal of using language for communication. The dimensions within each principle focus on methodological strategies for implementation. These strategies will be sensitive to the specific context in which we teach.

## Conclusion

This article has proposed a framework for language pedagogy which, though simple, is also faithful to the essential nature of learning and communication. In the classroom, its principles and dimensions may be implemented through strategies which are appropriate to specific contexts and also correspond to each teacher’s “sense of plausibility” based on experience (Maley, 2019; Prabhu, 2019). The authors hope that the article will be helpful in suggesting ways forward as we negotiate the waters of post-method language pedagogy.

## Data Availability Statement

The original contributions presented in the study are included in the article/supplementary material, and further inquiries can be directed to the corresponding author.

## Author Contributions

All authors listed have made a substantial, direct, and intellectual contribution to the work and approved it for publication.

## Conflict of Interest

The authors declare that the research was conducted in the absence of any commercial or financial relationships that could be construed as a potential conflict of interest.

## Publisher’s Note

All claims expressed in this article are solely those of the authors and do not necessarily represent those of their affiliated organizations, or those of the publisher, the editors and the reviewers. Any product that may be evaluated in this article, or claim that may be made by its manufacturer, is not guaranteed or endorsed by the publisher.
